# One-year follow-up for the therapeutic efficacy of pregabalin in patients with leg symptoms caused by lumbar spinal stenosis

**DOI:** 10.1007/s00776-014-0642-z

**Published:** 2014-10-22

**Authors:** Naoto Takahashi, Itaru Arai, Satoru Kayama, Kenji Ichiji, Hironari Fukuda, Shin-ichi Konno

**Affiliations:** 1 Department of Orthopaedic Surgery, Southern Tohoku General Hospital, Fukushima, Japan; 2 Department of Orthopaedic Surgery, Fukushima Medical University School of Medicine, 1 Hikarigaoka, Fukushima, 960-1295 Japan

## Abstract

**Background:**

Pregabalin is a well-accepted treatment option for patients with neuropathic pain. However, the therapeutic efficacy of pregabalin for reducing the incidence of spinal surgery to treat leg symptoms in patients with lumbar spinal stenosis remains unknown. The purpose of this study was to analyze the therapeutic efficacy of pregabalin for reducing the incidence of spinal surgery for leg symptoms in patients with lumbar spinal stenosis during the first year of treatment.

**Methods:**

Consecutive patients diagnosed with lumbar spinal stenosis at our hospital from January to June 2009 were treated with nonsteroidal anti-inflammatory drug monotherapy and formed the control group (*n* = 47; 22 males, 25 females). Patients diagnosed with lumbar spinal stenosis at our hospital between August 2010 and October 2011 were treated with a nonsteroidal anti-inflammatory drug and pregabalin combination therapy and formed the pregabalin group (*n* = 49; 27 males, 22 females). The proportions of patients who underwent spinal surgery during the first year of treatment were assessed and compared between the two groups using the Mann-Whitney *U* test. In addition, the periods in which patients decided to undergo spinal surgery were compared using the Kaplan-Meier method.

**Results:**

Six patients (12.2 %) in the pregabalin group and 22 patients (46.8 %) in the control group underwent spinal surgery during the first year of treatment (*P* = 0.0035). The period in which patients decided to undergo spinal surgery was significantly delayed in the pregabalin group compared with the control group in those for whom spinal surgery was necessary (*P* = 0.0128).

**Conclusions:**

Nonsteroidal anti-inflammatory drug and pregabalin combination therapy may result in a lower incidence of spinal surgery during the first year of treatment or a delayed period before undergoing spinal surgery if necessary compared with nonsteroidal anti-inflammatory drug monotherapy in patients with leg symptoms caused by lumbar spinal stenosis.

## Introduction

Lumbar spinal stenosis (LSS) is the most common reason for spinal surgery in patients older than 65 years [1, 2]. The symptoms of LSS may occur as a result of neurovascular mechanisms [3–5], such as reduced arterial flow in the cauda equina, venous congestion, increased epidural pressure, nerve root infiltration, and direct compression in the central canal or lateral recess [6]. The characteristic symptom of LSS is neurogenic intermittent claudication (NIC) [7, 8], but additional symptoms can include radicular pain down the leg, and numbness and motor weakness in the legs. If conservative treatments fail to improve the symptoms after 3–6 months, decompressive surgery is usually considered. However, patients aged above 65 years with symptomatic LSS have increased risks of complications in spinal surgery because of their previous illnesses and common comorbidities such as cardiovascular disease and chronic lung disease.

Pregabalin is a well-accepted treatment option for neuropathic pain owing to its analgesic, anxiolytic, and antiepileptic properties [9–11]. It is a structural analog of gamma-aminobutyric acid that potently and selectively binds to the alpha_2_-delta subunit of voltage-dependent calcium channels. Potent binding at these sites reduces the calcium influx at nerve terminals, thereby reducing the release of several excitatory neurotransmitters, including glutamate, noradrenaline, and substance P, and accounting for the therapeutic effects. Nonsteroidal anti-inflammatory drug (NSAID) and pregabalin combination therapy is one conservative treatment for LSS. However, it remains unknown whether NSAID and pregabalin combination therapy can have beneficial effects over a long period of time. The purpose of this study was to compare the incidences of spinal surgery during the first year of NSAID monotherapy and the first year of NSAID and pregabalin combination therapy.

## Materials and methods

We performed a retrospective cohort study of patients diagnosed with LSS at our institute between January 2009 and October 2011. The study received institutional review board approval from our hospital. Consecutive patients newly diagnosed with LSS from January to June 2009 were treated with NSAID monotherapy and formed the control group. Patients newly diagnosed with LSS between August 2010 and October 2011 were treated with NSAID and pregabalin combination therapy and formed the pregabalin group. The patients in the control group were not given pregabalin because the practice of prescribing pregabalin for patients with neuropathic pain was not adopted in Japan until August 2010. Three spinal surgeons diagnosed the patients with LSS, based not only on imaging findings of lumbar spinal canal stenosis, but also on subjective symptoms and/or neurological findings. Therefore, all patients had subjective symptoms and neurological findings caused by LSS, and imaging findings of lumbar spinal canal stenosis.

An independent radiologist assessed the magnetic resonance images obtained for each patient at the time of diagnosis for evidence of lumbar canal stenosis, which included central stenosis, lateral recess stenosis, and foraminal stenosis. The ankle brachial pressure index (ABI) was also checked in all patients to distinguish NIC from vascular intermittent claudication (ABI: <0.9). When providing informed consent, the patients were informed that surgical treatment was superior to conservative management of LSS [14–17] and that the therapeutic approach at our hospital was to use conservative management, reserving surgery and other therapies, such as epidural block or root block, for cases in which the therapeutic effect is insufficient during the first 3 months of conservative management. In the first 3 months of the study, patients who felt that the medical treatment was insufficient were able to request spinal surgical treatment. No patients reported wanting surgical or other therapies during this time period.

The inclusion criteria for all subjects were: (1) diagnosis of lumbar spondylosis or degenerative spondylolisthesis with LSS; (2) pain and/or numbness in the lumbar dermatomal distribution; (3) motor or sensory neurological signs (hypoesthesia, hyperesthesia, allodynia, or dysesthesia) in the affected dermatomes; (4) cognitive capability to complete our enquiries; (5) no previous history of treatment for symptoms of LSS; and (6) NIC caused by LSS. The exclusion criteria for all subjects were: (1) diagnosis of lumbar degenerative disease without LSS; (2) predominantly axial spinal pain; (3) significant motor deficits and/or bowel or bladder dysfunction; (4) rheumatoid arthritis; (5) known renal insufficiency, diabetes, congestive heart failure, cardiac conduction abnormalities, or thrombocytopenia; (6) known peripheral neuropathy; (7) history of spinal surgery; (8) history of workmen’s compensation or disability issues; (9) chronic depression and use of antidepressant medication; (10) renal dysfunction (creatinine clearance: <60 ml/min); (11) absolute requirement for surgical treatment because of tertiary paralysis or bladder dysfunction; and (12) ABI of <0.9. Additional exclusion criteria for the pregabalin group were: (1) previous history of gabapentin use or failure to respond to gabapentin use; (2) history of angioedema with pregabalin use; (3) known hypersensitivity to pregabalin use (hives, blisters, rash, dyspnea, or wheezing); and (4) need to drive a motor vehicle.

**Fig. 1:**
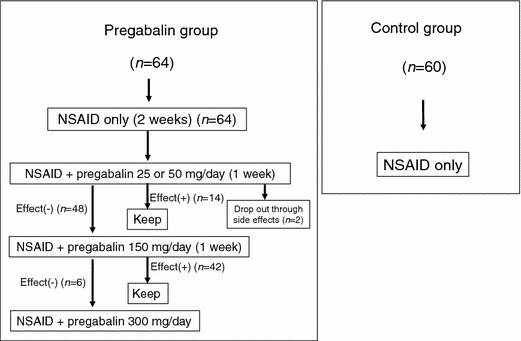
Flowchart of dosages in the pregabalin and control groups*. NSAID* Nonsteroidal anti-inflammatory drug

A total of 60 consecutive patients (30 male, 30 female) who were newly diagnosed with LSS at our hospital from January to June 2009 satisfied the inclusion criteria, did not meet the exclusion criteria, and provided informed consent for the treatment (Fig. 1). These patients were prescribed an NSAID, in the form of loxoprofen sodium hydrate or celecoxib. Within the first year after the start of medical treatment, eight patients were diagnosed with another disease, two patients died, one of a heart attack and the other of a malignant tumor, and three patients stopped the medical treatment and withdrew from all treatment. The remaining 47 patients (71.7 %; 22 males, 25 females) formed the control group. Thirty-one of these patients received loxoprofen sodium hydrate and 16 received celecoxib.

Among 126 patients who were newly diagnosed with LSS at our hospital from August 2010 to October 2011, 64 (30 male, 34 female) satisfied the inclusion criteria, did not meet the exclusion criteria and provided informed consent for the treatment. These patients were prescribed an NSAID, in the form of loxoprofen sodium hydrate or celecoxib, for the first 2 weeks, and pregabalin was added to the treatment regimen from the third week onward (Fig. 1). Pregabalin was started at a dose of 25 or 50 mg/day (Fig. 1). Patients with a body weight of ≥50 kg received a dose of 50 mg/day, while those with a body weight of <50 kg received a dose of 25 mg/day. If the selected dose did not produce sufficient pain relief within the first week, it was increased to 150 mg/day, and if that dose did not produce sufficient pain relief within the following week, it was further increased to 300 mg/day (Fig. 1). Pregabalin therapy was only started after renal function had been assessed to ensure that the creatinine clearance was >60 ml/min. Within the first year after the start of medical treatment, eight patients were diagnosed with another disease, one patient died because of a malignant tumor, and four patients stopped the medical treatment and withdrew from all treatment. Two of the 64 patients dropped out after experiencing side effects of pregabalin during treatment. The reported side effects of pregabalin were staggering, dizziness, and drowsiness. The remaining 49 patients (79 %; 27 males, 22 females) formed the pregabalin group. Thirty-one of these patients received loxoprofen sodium hydrate and 18 received celecoxib.

All patients were observed for the appearance of heart disease and intestinal hemorrhage, as well as disorders of internal organs such as the liver and kidney, while receiving NSAID treatment. All patients took a proton pump inhibitor once daily to prevent gastritis and/or gastroesophageal reflux disease. All patients were monitored throughout the year to determine whether they required spinal surgery. Absolute indications for spinal surgery were rarefied paralysis, dysfunction of bladder and bowel, or NIC within 10 min.

The numerical rating scale (NRS) score and Roland-Morris disability questionnaire (RDQ) score were used to compare the severity of subjective symptoms and quality of life associated with low back pain between the two groups before treatment. The NRS was used by the patients themselves for self-evaluation of their leg pain and/or numbness. The NRS and RDQ scores were examined before and after 3 months of treatment.

The primary outcome was the need for spinal surgery within 1 year after the start of medical treatment. We compared the demographic and clinical characteristics of the patients, the NRS and RDQ scores before and after 3 months of treatment, the distances causing NIC (<100 m, 100–500 m, or >500 m) before and after 3 months of treatment, and the proportions of patients who underwent spinal surgery during this time period between the two groups using the Mann-Whitney U test. In addition, the periods in which the patients decided to undergo spinal surgery, if necessary, were compared between the two groups using the Kaplan-Meier method. Values of *P* < 0.05 were considered to indicate statistical significance. All statistical analyses were performed using StatView 5.0 statistical software (SAS Inc., Cary, NC).

## Results

The demographic and clinical characteristics of the patients are shown in Table 1. The age, sex distribution, physical status, smoking status, proportion of patients performing manual labor, and proportion of patients with professional qualifications were similar between the pregabalin and control groups. In both groups, the majority of patients were aged between 60 and 70 years. The mean duration of pain, affected spinal level, number of spinal stenosis levels on magnetic resonance images, cause of LSS, and NRS and RDQ scores before treatment (Tables 2 and 3) were also similar between the pregabalin and control groups. However, the NRS (*P* < 0.0001) and RDQ (*P* = 0.0001) scores after 3 months of treatment were significantly lower in the pregabalin group than in the control group (Tables 2 and 3). The distances causing NIC (<100 m, 100–500 m, or >500 m) before and after 3 months of treatment were similar between the pregabalin and control groups (Table 4).

**Table 1:** Demographic and clinical characteristics of the patients

	Pregabalin group (*n* = 49)	Control group (*n* = 47)	*P* value
**Age (years)**	68.1 ± 1.56	68.5 ± 1.48	0.983^a^
**Sex**			0.484^a^
Male	27 (55.1)	22 (46.8)	
Female	22 (44.9)	25 (53.2)	
**American Society of Anesthesiologists physical status**			0.997^a^
Level I	25 (51.0)	24 (51.1)	
Level II	24 (49.0)	23 (48.9)	
**Current smoker**	4 (8.1)	3 (6.4)	0.881^a^
**Manual laborer**	13 (26.5)	12 (25.5)	0.933^a^
**Professional qualification (s)**	10 (20.4)	9 (19.1)	0.915^a^
**Duration of pain, months**	23.0 ± 6.19	26.4 ± 8.48	0.745^a^
**Affected spinal level**			0.889^a^
L3–L4	5 (10.2)	5 (10.6)	
L4–L5	42 (85.7)	39 (83.0)	
L5–S1	2 (4.1)	3 (6.4)	
**Number of spinal stenosis levels on magnetic resonance images**			0.679^a^
One level	30 (61.2)	31 (66.0)	
Two levels	14 (28.6)	12 (25.5)	
More than three levels	5 (10.2)	4 (8.5)	
**Cause of lumbar spinal stenosis**			0.129^a^
Lumbar spondylitis	38 (77.6)	29 (61.7)	
Degenerative spondylolisthesis	11 (22.4)	18 (38.3)	

Data are shown as mean ± standard error or *n* (%)

^a^Mann-Whitney *U* test

**Table 2:** NRS scores before and after 3 months of treatment in the two groups

	Pregabalin group (*n* = 49)	Control group (*n* = 47)	*P* value
NRS score before treatment	7.98 ± 0.25	8.15 ± 0.18	0.717^a^
NRS score after 3 months of treatment	2.90 ± 0.32	5.91 ± 0.37	<0.0001^a^

Data are shown as mean ± standard error or *n* (%)

^a^Mann-Whitney *U* test

**Table 3:** RDQ scores before and after 3 months of treatment in the two groups

	Pregabalin group (*n* = 49)	Control group (*n* = 47)	*P* value
RDQ score before treatment	16.0 ± 0.86	17.0 ± 0.74	0.439^a^
RDQ score after 3 months of treatment	6.84 ± 1.26	12.3 ± 1.09	0.0001^a^

Data are shown as mean ± standard error or *n* (%)

^a^Mann-Whitney *U* test

**Table 4:** Distances causing NIC before and after 3 months of treatment in the two groups

	Pregabalin group (*n* = 49)	Control group (*n* = 47)	*P* value
Distance causing NIC before treatment (<100 m/100–500 m/>500 m)	11/32/6	10/27/10	0.50^a^
Distance causing NIC after 3 months of treatment (<100 m/100–500 m/>500 m)	7/26/16	9/23/15	0.98^a^

Data are shown as mean ± standard error or *n*

*NRS* Numerical rating scale, *RDQ* Roland-Morris disability questionnaire, *NIC* neurogenic intermittent claudication

^a^Mann-Whitney *U* test

No patients underwent spinal surgery during the first 3 months of medical treatment. Of the 49 patients in the pregabalin group, 4 (2 males, 2 females) recovered within the first year of medical treatment, 6 (2 males, 4 females) required spinal surgery within the first year of medical treatment, and 39 (23 males, 16 females) continued the medical treatment after 1 year. Of the 47 patients in the control group, 4 (3 males, 1 female) recovered after the first year of medical treatment, 22 (9 males, 13 females) required spinal surgery treatment within the first year of medical treatment, and 21 (10 males, 11 females) continued the medical treatment after 1 year. Thus, 6 of 49 patients (12.2 %) in the pregabalin group and 22 of 47 patients (46.8 %) in the control group required spinal surgery treatment between 3 months and 1 year after the start of medical treatment. These proportions differed significantly between the two groups (*P* = 0.0035). The reasons for the spinal surgeries are shown in Table 5. All patients in the pregabalin and control groups required spinal surgery because of insufficient effects of medical treatment on pain reduction. Two of 6 patients (33.3 %) who required surgery in the pregabalin group and 10 of 22 patients (45.5 %) who required surgery in the control group had aggravation of NIC.

**Table 5:** Reasons for spinal surgeries in the two groups

	Pregabalin group (*n* = 6)	Control group (*n* = 22)
Insufficient pain reduction by pharmacotherapy	6	22
Aggravation of NIC	2	10

*NIC* Neurogenic intermittent claudication

The period in which the patients decided to undergo spinal surgery was significantly delayed in the pregabalin group compared with the control group in those for whom spinal surgery was necessary (*P* = 0.0128) (Table 6).

**Table 6:** Period when the patients decided to have the spinal surgery

	Pregabalin group (*n* = 6)	Control group (*n* = 22)	*P* value
The period when the patients decided to have spinal surgery	6.67 ± 1.23	5.59 ± 0.813	0.0128^a^

Data are shown as mean ± standard error or *N* (%)

^a^Kaplan-Meier method

## Discussion

The present study has demonstrated that (1) the NRS and RDQ scores before treatment were similar between the pregabalin and control groups, while those after 3 months of treatment were significantly lower in the pregabalin group than in the control group; (2) the distances causing NIC (<100 m, 100–500 m, or >500 m) before and after 3 months of treatment were similar between the two groups; and (3) the period in which the patients decided to undergo spinal surgery was significantly delayed in the pregabalin group compared with the control group in those for whom spinal surgery was necessary. These data suggested that the incidence of spinal surgery within the first year of medical treatment for LSS was significantly lower among patients who received NSAID and pregabalin combination therapy than among patients who received NSAID monotherapy.

LSS may occur at different levels in the spinal canal and may occur at more than one level at the same time. Central canal stenosis may compress nerve roots in the cauda equina, whereas lateral recess stenosis and foraminal stenosis may compress nerve roots while sparing the spine [18, 19]. Although the lower limb symptoms of LSS are mainly attributed to mechanoreceptive compression of nerve rootlets and the cauda equina, they are also associated with inflammation, ischemia, malnutrition, nerve degeneration, and nerve injury and consequently have a complicated pathophysiology. Therefore, it may be demonstrated that the pathomechanisms of lower limb symptoms caused by LSS involve nociceptive, inflammatory and/or neuropathic pain components. These can result from postural changes or persistent compression of the nerve roots and/or cauda equina while walking.

Pregabalin is effective at reducing neuropathic pain [10–13], but may have few therapeutic effects on inflammatory and nociceptive pain. Recent studies using the pain DETECT screening questionnaire [20] demonstrated that the neuropathic component of pain was more intense than the other components of pain in patients with chronic lower back pain [20] and that pain, disability, anxiety, and depression were higher, and quality of life and range of motion for passive straight leg raising were lower in patients with neuropathic back and leg pain than in patients with nociceptive back and leg pain [20, 21]. These results suggest that pregabalin may be effective in LSS patients with radicular pain, particularly neuropathic radicular pain. The results of Takahashi et al. [12] support this hypothesis, as they found that NSAID and pregabalin combination therapy was more effective for relief of leg symptoms than NSAID monotherapy in the chronic phase of LSS over 3 months after the first appearance of symptoms and prevented aggravation of self-reported symptoms in patients with radicular- and mixed-type NIC [12]. Therefore, the results of the present study may support the hypothesis that NSAID and pregabalin combination therapy can reduce the incidence of spinal surgery for patients with leg symptoms caused by LSS. This may be beneficial, especially for older patients who are at high risk for complications after spinal surgery.

Generally, surgical treatment is superior to conservative treatment for LSS irrespective of the degree of affectation and whether the patient has spondylolisthesis or NIC [14–17]. Spinal surgery can be effective despite advanced age, multilevel involvement, and comorbidities such as diabetes, obesity, chronic coronary disease, and chronic lung disease [17, 22–24]. However, these features, especially chronic coronary disease and chronic lung disease as well as hospitalization within the year prior to surgery are associated with increased complications and mortality [24]. Therefore, the risks of spinal surgery should be balanced against expected improvements for individual patients.

The present study has some limitations that require attention. First, the follow-up period was relatively short, and future studies are required to evaluate the long-term therapeutic efficacy of NSAID and pregabalin combination therapy. Second, we did not evaluate the therapeutic efficacy of pregabalin alone, and future studies are required to evaluate the therapeutic efficacy of pregabalin monotherapy. Third, this was a retrospective cohort study and therefore open to selection bias. Fourth, two different NSAIDs were used. A future study with standardization of the NSAID treatment is necessary. A clinical study with postlicensure surveillance should be implemented, ideally by setting up a database that includes all patients seeking treatment for leg symptoms caused by LSS, minimizing losses to follow-up, and using validated methods to gather clinically relevant data including demographic information, clinical features, common comorbidities, conservative and surgical treatments applied to each patient, experience and training standards of the care providers applying each treatment, and each patient’s clinical evolution [25].

In conclusion, LSS patients who received NSAID and pregabalin combination therapy had a lower incidence of spinal surgery for treatment of leg symptoms within the first year of therapy and a delayed period before undergoing spinal surgery if necessary compared with LSS patients who received NSAID monotherapy. NSAID and pregabalin combination therapy may be particularly useful for patients of advanced age, who are at high risk of complications from spinal surgery.
